# A New Pyranoxanthone from *Calophyllum soulattri*

**DOI:** 10.3390/molecules16053999

**Published:** 2011-05-12

**Authors:** Siau Hui Mah, Gwendoline Cheng Lian Ee, Mawardi Rahmani, Yun Hin Taufiq-Yap, Mohd Aspollah Sukari, Soek Sin Teh

**Affiliations:** Department of Chemistry, Faculty of Science, Universiti Putra Malaysia, 43400 UPM Serdang, Selangor, Malaysia

**Keywords:** soulattrin, pyranoxanthone, *Calophyllum soulattri*, Guttiferae

## Abstract

Our interest on xanthones from the *Calophyllum* genus has led us toa detailed study on the chemistry of the stem bark of *Calophyllum soulattri.* This gave one new pyranoxanthone, soulattrin (**1**), together with three other xanthones, caloxanthone B (**2**), caloxanthone C (**3**), macluraxanthone (**4**), the triterpene friedelin (**5**) and the steroid stigmasterol (**6**). The identities of these compounds were established using analyses of 1D and 2D NMR data.

## 1. Introduction

*Calophyllum* is widely distributed in Asia, Australia, Africa and Polynesia. Some species of *Calophyllum* have been used in folk medicine. Previous studies indicated the existence of a variety of secondary metabolites such as xanthones [1], coumarins [2], triterpenoids [3] and flavonoids [4,5] which possess various bioactivities. In 1998 *Calophyllum* was found to possess anti-HIV activity [6,7]. Some of these biologically active compounds have also shown antifungal [8], antimicrobial [9] and molluscicidal [10] effects. Natural products from *Calophyllum* are also well recognized by the industry as cancer chemo-preventive agents [11]. This work concentrated on the isolation and structural elucidation of the new xanthone soulattrin (**1**) from the stem bark of *Calophyllum soulattri*.

## 2. Results and Discussion

Compound **1** was isolated as yellow crystals with melting point 180–181 °C. EIMS gave a molecular ion peak at *m/z* 394, while the HRESIMS spectrum gave 394.1550 (calc’d 394.1417) which is consistent with the molecular formula C_23_H_22_O_6_. The FTIR spectrum gave absorptions of free hydroxyl (3,300 cm^−1^), conjugated carbonyl (1,640 cm^−1^) and aromatic ring (1,583 cm^−1^), which reflected similarity to typical IR bands for xanthones. Maximum absorptions of UV spectrum were observed at 336, 291, 281 and 242 nm, also suggesting a xanthone skeleton.

^13^C-NMR and DEPT spectra revealed one carbonyl carbon at δ 181.1 and twelve other quaternary carbons, seven of which (δ 78.2, 133.0, 146.0, 151.2, 155.3, 156.5 and 158.8) are oxygenated. Meanwhile, the non-oxygenated quaternary carbons resonated at δ 41.0, 102.8, 104.9 × 2 and 113.5. Besides this, five methine carbon signals (δ 112.9, 115.6, 116.2, 127.5 and 151.8), one methylene signal (δ 106.7) and four methyl carbon signals (δ 27.2 × 2 and 29.3 × 2) were also observed in the ^13^C-NMR and DEPT spectra. 

**Figure 1:**
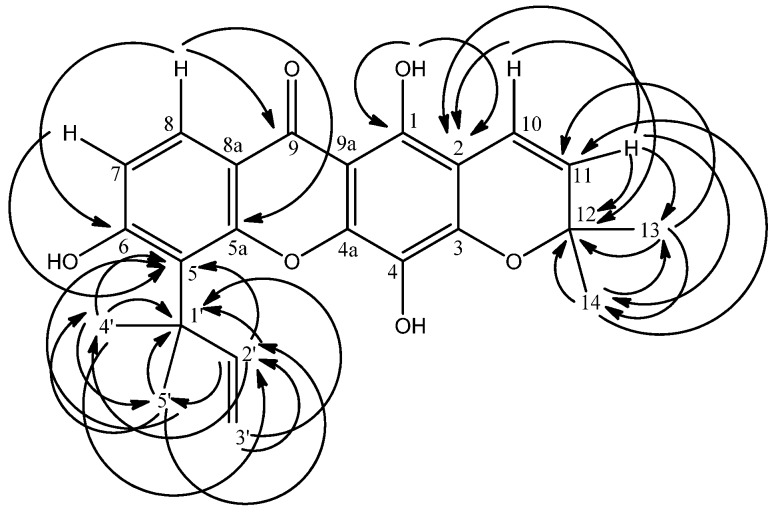
HMBC^ 2^*J* and ^3^*J* correlations between ^1^H and ^13^C in **1**.

A prenyl group with its characteristic chemical shifts was also seen in the ^1^H-NMR spectrum of **1**. The ^1^H resonances occurred at δ 1.70 × 2 (s, 3H, H-4’ & 5’), 4.84 (d, 1H, *J* = 10.3 Hz, H-3’b), 4.99 (d, 1H, *J* = 18.3 Hz, H-3’a) and 6.46 (dd, 1H, *J* = 10.3 Hz & 18.3 Hz, H-2’), while the carbon resonances occurred at δ 29.3 × 2 (C-4’ & 5’), 41.0 (C-1’), 106.7 (C-3’a & 3’b) and 151.8 (C-2’). Further confirmation was achieved through long range correlations between the two vinylic methyl protons at δ 6.46 (H-2’) and 4.84 and 4.99 (H-3’b & 3’a) and their neighbouring carbon at δ 41.0 (C-1’). The two methyl signals (δ 1.70 × 2) also showed ^2^*J* and ^3^*J* correlation with δ 41.0 (C-1’) and 151.8 (C-2’). Placement of the prenyl group at C-5 was achieved via observations of ^3^*J* correlations between H-2’ (δ 6.46), H-4’ & 5’ (δ 1.70 × 2) and C-5 (δ 113.5) (Figure 1).

**Figure 2:**
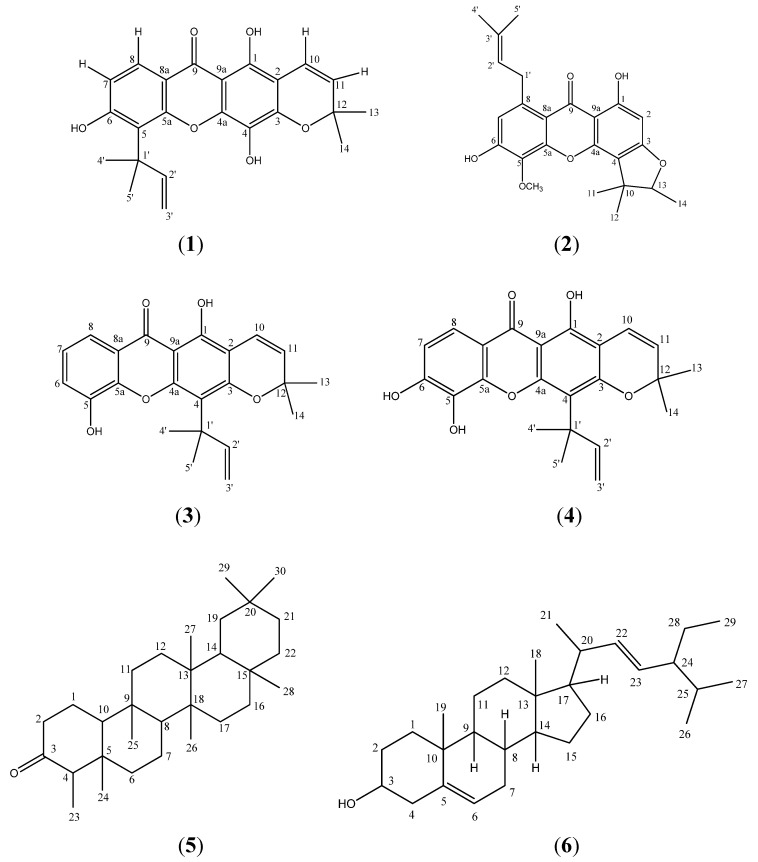
Structures of soulattrin (**1**), caloxanthone B (**2**), caloxanthone C (**3**), macluraxanthone (**4**), friedelin (**5**) and stigmasterol (**6**).

The ^1^H-NMR spectrum of **1** also revealed the presence of one pyrano ring. One pair of *ortho-* coupled protons were observed at δ 5.64 (d, 1H, *J* = 9.8 Hz) and 6.66 (d, 1H, *J* = 9.8 Hz) for H-11 and H-10. Meanwhile, one pair of overlapping methyls gave a signal at δ 1.45 (s, 6H, H-13 & 14). This signal at δ 1.45 gave cross peaks to the carbon signal at δ 127.5 (C-11, ^3^*J*) and 78.2 (C-12, ^2^*J*) indicating the two methyls to be directly bonded to C-12 which is in turn bonded to C-11, implying the presence of a pyrano ring. The doublet signal at δ 5.64 (H-11) and 6.66 (H-10) gave a ^3^*J* correlation to the carbon signal at δ 104.9 (C-2). Hence the pyrano ring was assigned to C-2 and C-3. The ^13^C-NMR spectrum also gave a quaternary carbon at δ 78.2 (C-12) which further supports the existence of this pyrano ring. One sharp singlet was observed in the ^1^H-NMR spectrum at δ 6.24. The chelated OH signal (δ 13.86) gave cross peaks with δ 104.9 (C-2, ^3^*J*) and 156.5 (C-1, ^2^*J*) allowing its placement at C-1. On the basis of the above interpretation of the 1D and 2D NMR data, the structure of compound **1 **was elucidated to be 1,4,6-trihydroxy-5-(1’,1’-dimethyl-2’-propenyl)-6”,6”-dimethylpyrano-[2”,3”:3,2]xanthone and named soulattrin (**1**, Figure 1). Spectral data for compounds **2-6** (Figure 2) were in agreement with published data.

## 3. Experimental

### 3.1. Plant Material

The stem bark of *Calophyllum soulattri* was collected from the Sri Aman district in Sarawak, Malaysia by Dr. Jegak Uli. This plant was identified by Dr. Rusea Go from the Department of Biology, Faculty of Science, Universiti Putra Malaysia. 

### 3.2. General

EIMS were recorded on a Shimadzu GC-MS model QP2010 Plus spectrophotometer. NMR spectra were obtained using a JEOL FTNMR 400 MHz spectrophotometer using tetramethylsilane (TMS) as internal standard. Ultraviolet spectra were recorded in EtOH on a Shimadzu UV-160A UV-Visible Recording Spectrophotometer. Infrared spectra were measured using the universal attenuated total reflection (UATR) technique on a Perkin-Elmer 100 Series FT-IR spectrometer. Melting points were measured using Leica Galen III microscope, equipped with Testo 720 temperature recorder.

### 3.3. Extraction and Isolation

Approximately 1 kg of air-dried stem bark of *Calophyllum soulattri* was ground into fine powder and extracted successively in a Soxhlet apparatus with *n*-hexane, dichloromethane, ethyl acetate and methanol for 72 hours. The extracts were evaporated to dryness under vacuum to give 101.2 g of hexane extract and 15.3 g of dichloromethane extract. Part of each extract was subjected to column chromatography over silica gel and eluted with a stepwise gradient system using *n*-hexane, dichloromethane, ethyl acetate and methanol. Further purifications of the hexane extract afforded friedelin (**5**, 450 mg). Meanwhile, purification of the dichloromethane extract afforded the new xanthone, soulattrin (**1**, 7 mg), caloxanthone B (**2**, 12 mg), caloxanthone C (**3**, 14 mg), macluraxanthone (**4**, 6 mg) and stigmasterol (**6**, 22 mg).

### 3.4. Spectral Data

*Soulattrin* (**1**) Yellow crystals; m.p. 180–181 °C; UV (EtOH) λ_max_ nm (log ε): 336 (5.42), 291 (5.36), 281 (5.35), 242 (5.28); IR ν_max_ cm^−^^1^: 3300, 2969, 1640, 1583; EIMS m/z (rel. int.): 394 [M^+^] (33), 379 (100), 365 (6), 339 (6), 321 (6), 182 (11), 162 (18); HRESIMS: 394.1550 (Calc’d. for C_28_H_28_O_6_: 394.1417); ^1^H-NMR (CDCl_3_): δ 13.86 (OH-1, s), 6.95 (1H, d, *J* = 9.2 Hz, H-7), 7.54 (1H, d, *J* = 9.2 Hz, H-8), 6.66 (1H, d, *J* = 9.8 Hz, H-10), 5.64 (1H, d, *J* = 9.8 Hz, H-11), 6.46 (1H, dd, *J* = 10.3 Hz & 18.3 Hz, H-2’), 4.99 (1H, d, *J* = 18.3 Hz, H-3’a), 4.84 (1H, d, *J* = 10.3 Hz, H-3’b), 1.70 (6H, s, H-4’ & H-5’), 1.45 (6H, s, H-13 & H-14); ^13^C-NMR (CDCl_3_): δ 181.1 (C-9), 158.8 (C-3), 156.5 (C-1), 155.3 (C-4a), 151.8 (C-2’), 151.2 (C-6), 146.0 (C-5a), 133.0 (C-4), 127.5 (C-11), 116.2 (C-8), 115.6 (C-10), 113.5 (C-5), 112.9 (C-7), 107.0 (C-3’), 104.9 (C-2 & C-9a), 102.8 (C-8a), 78.2 (C-12), 41.0 (C-1’), 151.8 (C-2’), 106.7 (C-3’), 29.3 (C-4’ & C-5’), 27.2 (C-13 & C-14).

*Caloxanthone* B (**2**) Yellow needles; m.p. 157–158 °C (Lit. 160.5 °C)[12]; Spectral data are consistent with literature [12].

*Caloxanthone* C (**3**) Yellow needles; m.p. 210–212 °C (Lit. 217 °C)[13]; spectral data are consistent with literature [13].

*Macluraxanthone* (**4**) Yellow needles; m.p. 181–182 °C (Lit. 170-172 °C)[12]; spectral data are consistent with literature [12].

*Friedelin* (**5**) White needles; m.p. 245–246 °C (Lit. 246–248 °C)[2]; spectral data are consistent with literature [2].

*Stigmasterol* (**6**) White needles; m.p. 155–157 °C (Lit. 168–169 °C)[15]; spectral data are consistent with literature [15].

## 4. Conclusions

The stem bark of *Calophyllum soulattri* furnished one new prenylated pyranoxanthone, soulattrin (**1**), together with three other xanthones, caloxanthone B (**2**), caloxanthone C (**3**) and macluraxanthone (**4**) the common triterpene friedelin (**5**) and the steroid stigmasterol (**6**).
